# Nondual Awareness and Minimal Phenomenal Experience

**DOI:** 10.3389/fpsyg.2020.02087

**Published:** 2020-08-21

**Authors:** Zoran Josipovic, Vladimir Miskovic

**Affiliations:** ^1^ Department of Psychology, New York University, New York City, NY, United States; ^2^ Department of Psychology, Binghamton University, Binghamton, NY, United States

**Keywords:** nondual awareness, minimal phenomenal experience, consciousness, meditation, lucid NREM sleep, consciousness-as-such

## Abstract

Minimal phenomenal experiences (MPEs) have recently gained attention in the fields of neuroscience and philosophy of mind. They can be thought of as episodes of greatly reduced or even absent phenomenal content together with a reduced level of arousal. It has also been proposed that MPEs are cases of consciousness-as-such. Here, we present a different perspective, that consciousness-as-such is first and foremost a type of awareness, that is, non-conceptual, non-propositional, and nondual, in other words, non-representational. This awareness is a unique kind and cannot be adequately specified by the two-dimensional model of consciousness as the arousal level plus the phenomenal content or by their mental representations. Thus, we suggest that to understand consciousness-as-such, and by extension consciousness in general, more accurately, we need to research it as a unique kind.

## Introduction

We have previously proposed that the impasse in today’s scientific understanding of consciousness would be helped by understanding consciousness itself or nondual awareness (NDA), a basic aspect of consciousness that is different from all other phenomenal contents and functions of consciousness and from global states of arousal (Josipovic, 2014, 2019; Josipovic and Baars, 2015). Attempts to include this topic within the realm of contemporary scientific and philosophical discourse, which have been halting in the past (Forman, 1998; Shear and Jevning, 1999; Travis and Pearson, 2000), have recently gained renewed traction in both neuroscience and philosophy of mind (Baars, 2013; Tononi and Koch, 2015; Windt, 2015; Dor-Ziderman et al., 2016; Milliere et al., 2018; Koch, 2019; Metzinger, 2019, 2020; Miskovic et al., 2019). In particular, it has been proposed that so-called minimal phenomenal experiences (MPEs) or full absorption states are examples of consciousness-as-such (Metzinger, 2019).

Consciousness-as-such has been present as a central concern in most Asian contemplative traditions and philosophies, where a great variety of terms have been created to point to it, such as pure consciousness, pure awareness, NDA, rigpa, timeless or choiceless awareness, being-awareness-bliss, the Self, the fourth, Atman-Brahman, Buddha-nature, clear light, Shiva-Shakti, etc. (Rabjam, 2007; Radhakrishnan and Moore, 2014). These are primarily concerned with phenomenal descriptions but may also contain elements of ontological and metaphysical inferences and various pedagogical, ethical, or soteriological concerns, which we will not discuss here.

Two main ways of seeing consciousness-as-such have emerged in these traditions. According to one, it is a state of full absorption with maximally reduced phenomenal content (Sansk. Samadhi), akin to a deep dreamless sleep, but not entirely unconscious, and without any knowable properties (Nikhilananda, 1990). According to the other, it is an awareness which is in itself empty of other phenomenal content and conceptual processes including those that reify it, yet it inherently knows itself to be aware (Rabjam, 2001). In the contemporary scientific and philosophical discourse, these two views are often conflated, sometimes under one term of MPE (Metzinger, 2019).

Here we argue that the two should not be conflated, and that the latter view, which understands consciousness-as-such as a unique kind of awareness, is more accurate and useful for guiding contemporary discourse on consciousness. Specifically, we show that the two-dimensional model of consciousness as the arousal level plus the experiential content does not adequately specify consciousness-as-such. In line with our previous work (Josipovic, 2014, 2019), we advance the perspective that consciousness-as-such is first and foremost a type of awareness, that is non-conceptual, non-propositional, and nondual, in other words, non-representational. This awareness is a unique kind, and cannot be reduced to a level of arousal and phenomenal content, or to their mental representations and representational models.

The term MPE, as originally used, refers to episodes of greatly reduced or minimal phenomenal content, accompanied by reduced levels of arousal (Windt, 2015). At times, the term has been greatly stretched to include many types of contemplative experiences with differing amounts and complexity of phenomenal contents and levels of arousal, and with different properties of NDA, so that the term has lost much of its original meaning, and appears to overlap and confound with the term NDA (Metzinger, 2020). We will stay with the original meaning of the term MPE, as referring to episodes of reduced or minimal phenomenal content and reduced arousal. During such MPE there may, or may not, be an explicitly present NDA, which indicates that the term NDA refers to something essentially different from such MPE.

Episodes of explicitly present NDA that is isolated from other phenomenal content, such as those that can occur with contemplative practice or in lucidity during deep sleep, can be regarded as episodes of MPE since no phenomenal content is present other than awareness itself (Josipovic, 2019). However, since NDA, as we will see, can be present with any amount of phenomenal content, the term MPE does not best capture what NDA actually is, a type of awareness. Furthermore, MPE has at times been equated with full absorption (Sansk. Samadhi), a term borrowed from contemplative traditions where it most often indicates a greatly reduced level of arousal accompanied by minimal or no phenomenal content (Aranya, 1984; Metzinger, 2020). However, there is often no clarity in the traditional accounts as to whether full absorption entails the presence of NDA or not and whether that awareness, if present, is self-recognized or not (Srinivasan, 2020). Hence, there is a frequent misunderstanding that a mere absorption with a lack of most content, also known as mental silence, is the same as NDA. Likewise, in the full waking state, a mere presence without noticeable thoughts or emotions is often mistaken for NDA (Rabjam and Thondup, 2002). These and other similar types of conflations and misunderstandings are based, at least in part, on the reluctance to view NDA as a unique kind, one that is not specified by the level of arousal or the amount of phenomenal content.

What makes consciousness-as-such or NDA a unique kind is that it is aware, and inherently knows itself to be aware, without relying on mental representations (Josipovic, 2019). The two most common fixed beliefs that make this difficult to understand are that all knowing has to be mediated mental representations, also known as strong representationalism, and that being a unique kind necessarily implies naive essentialism and substance dualism. The first belief has been argued against extensively by most nondual contemplative traditions, especially the Buddhist ones (Rabjam, 2007; Dunne, 2015; Josipovic, 2019), as well as, by the phenomenologists, as this well-known quote indicates: “The pre-reflective self-awareness of an experience is not mediated by foreign elements such as concepts and classificatory criteria, nor by any internal difference or distance. It is an immediate and direct self-acquaintance which is characterized by being completely and absolutely irrelational (and consequently best described as a purely immanent self-presence)” (Frank, as quoted in Zahavi, 1998). The second belief is based on a limited interpretation that being a unique kind is due only to conceptual categorization. It also conflates phenomenological and ontological levels of discourse. The claim that differentiating awareness and phenomenal contents creates an artificial duality (Bayne et al., 2016) does not apply to NDA, which, as we will see, though distinct, co-occurs with phenomena nondually (Rabjam, 2001; Josipovic, 2019).

Below, we give some examples and outline the main properties of experiences of reduced and minimal phenomenal content, and those of NDA, and then discuss their main differences. We show that NDA and MPE should be treated as distinct and not be conflated and that only NDA can truly be considered to be consciousness-as-such.

## Minimal Phenomenal Experience

Experiences of reduced phenomenal content can occur in several situations: naturally, when transitioning to and from sleep or under special circumstances, such as when waking from anesthesia, in some minimally conscious states and near-death experiences, during sensory deprivation, under the influence of mind-altering substances, and during meditation and related practices (Windt, 2015; Dor-Ziderman et al., 2016; Milliere et al., 2018; Metzinger, 2019, 2020; Miskovic et al., 2019). These experiences can have varying degrees of arousal or wakefulness, from low levels of arousal akin to deep sleep to high levels of hyper-arousal accompanying some altered states. Likewise, the amount and type of phenomenal content can vary greatly, and reductions or even absences of the perception of time, space, body, and self-have been reported (Dor-Ziderman et al., 2016; Metzinger, 2020). Additionally, the degree of consciousness can also vary, from a complete absence in various types of blackouts on one end of the spectrum, which may preclude such events from being regarded as phenomenal experiences altogether, to a vivid presence of self-knowing luminous awareness, regarded in some cases as instances of consciousness directly encountering itself (Josipovic, 2019).

More recently, Windt (2015) introduced the term MPE to refer to a special class of phenomenal experience with reduced content, in which content is reduced to what can arguably be regarded as some minimum that satisfies specific, albeit differing, constraints and lacks all perspectival structures (see also Forman, 1998 and Metzinger, 2019). While such MPEs are characterized by absence of most phenomenal content, including any egoic self-consciousness, one is not simply unconscious as in deep sleep (Windt et al., 2016). However, what exactly is left in such experiences is not entirely clear, neither in the traditional accounts, nor in the contemporary discourse (Srinivasan, 2020). This becomes especially problematic when the term MPE is stretched and applied to a broad range of experiences with varying degrees of phenomenal content and levels of arousal, so that the term itself loses its intended meaning (Metzinger, 2020). Therefore, presently we will stay with the original meaning of the term as stated above, so that MPE here refers to experiences of greatly reduced or minimal phenomenal content that are also accompanied with lowered levels of arousal, such as those occurring during initial stages of lucidity in deep sleep or during the full absorption in meditation. Importantly, whether NDA is explicitly present in MPEs or not is orthogonal to such MPEs.

While describing all or even most types of experiences of reduced or minimal phenomenal content is outside of the scope of this paper, below we describe briefly a few cases related to sleep and to meditation practice, as the most obvious examples of either naturally occurring or induced types, with reduced or minimal phenomenal content. Their common characteristic is that they can be specified by the amount of residual phenomenal content and by the level of arousal. In contrast, as we will see, NDA, whether explicitly present or not in these experiences, is independent from both the amount and complexity of phenomenal content, and from the level of arousal, and is not specified by them.

### Reduced and Minimal Phenomenal Content in Sleep

The transformations of consciousness that are naturally observed across distinct sleep stages, including both non-rapid eye movement (NREM) and REM sleep, provide the most readily available opportunity for studying phenomenal experiences with reduced or even minimal content. An early reference to this phenomenon can be found in the writings of the psychoanalyst, Lewin (1946), who drew attention to what he called the blank dream. These were dreams corresponding to an experience of the empty visual screen onto which the manifested elements of the dream are projected. While the screen itself is not usually seen as a distinct element (attention being captivated by the dream narrative unfolding in dream space), not so in blank dreams where the screen appears alone.

The psychoanalytic discovery of the blank dream went mostly unnoticed by mainstream psychologists conducting experimental research on sleep and dreaming. As often happens, however, a related phenomenon was independently noted about three decades later, when it was discovered that around 30% of dreams in carefully collected diaries were lacking in any content (Cohen, 1972) – so-called “white dreams” (Fazekas et al., 2019). This finding has been supported by research using the serial awakening paradigm, wherein participants were woken up at regular 15–30 min intervals (Siclari et al., 2013). By comparison, another 20% of the awakenings were followed by descriptions in which there was no sense of any kind of dreaming having taken place, only a vacuity, while the remainder consisted of narrative rich dreams. Using early night-serial awakenings, Noreika et al. (2009) found that the frequency of white dreams was close to 40% during Stages 2 and 3 of NREM sleep. Fragmentary instances of white dreaming can also sometimes be reported during sedation with general anesthetics in cases where participants were no longer responsive to behavioral commands (Noreika et al., 2011).

While it is certainly plausible that many white dreams reflect memory failures rather than genuine contentless experiences *per se* (Siclari et al., 2017), others have argued that (a) white dreams do indeed contain some content, but that (b) the content is of a weak perceptual quality that lies between the vividness of a typical dream and the experience of deep, dreamless sleep (Fazekas et al., 2019). Support for this hypothesis comes from an EEG study (Siclari et al., 2017) which reported that, compared to no dreaming and dreams with recalled content, white dreams are associated with intermediate power in high frequency oscillations recorded from sensory “hot zones.” Reduced neural activity in posterior regions may correspond to perceptual representations occupying the low quality end of the spectrum of conscious experiences, characterized by diminished vividness, greater ambiguity, and less stability (Fazekas et al., 2019).

### Lucidity in Dreamless Sleep

Whether white dreams are best accounted for by degraded perceptual content or by the near absence of content altogether remains unclear, but the latter possibility is attested to by imageless lucid dreaming (Windt, 2015). These are dreams in which a minimal form of presence is maintained (although this presence can be completely disembodied), in the apparent absence of any sensory images. One account describes this state as one in which “no symbols are encountered, visual or otherwise… all awareness of the self as body or special entity leaves… characterized by peace, silence…” (Magallón, 1991; see also LaBerge and DeGracia, 2000; Bogzaran, 2003).

Since spontaneous or state induced MPEs are transient and highly unstable events, their recognition by untrained observers is extremely difficult and rare. Not surprisingly, advanced meditators, who have developed the requisite stability of attention, have provided us the largest amount of data on these conscious states. For example, sleep yoga practices developed in the context of the Tibetan Buddhist tradition allow the dedicated practitioner the ability to enter into “clear light sleep” - a form of dreamless sleep that is characterized not by mental blankness and sluggishness, but a high degree of clarity, described as: “…there is no film and no projection. Sleep yoga is imageless. The practice is the direct recognition of awareness by awareness, light illuminating light itself. It is luminosity without images of any kind” (Wangyal, 1998, p.). Such episodes can be regarded as episodes of NDA co-occurring with minimal phenomenal content, though not all lucidity in deep sleep is necessarily NDA.

### Meditative Absorption States

Meditative absorption states are reported as states of progressive decrease of phenomenal content and arousal that are induced through applying various cognitive strategies, mainly involving attention regulation, working memory, and reward prediction (Lutz et al., 2008; Josipovic, 2010; Josipovic et al., 2012). Different meditation techniques and different stages of the same technique can rely predominantly on one or the other function, such as top-down voluntary sustained attention, bottom-up involuntary salience-driven attention, open monitoring and vigilance, or some combination of these. Different degrees of effort are applied, usually decreasing with developing skill levels, though some techniques insist on a minimal effort from the start (Wallace, 2006; Travis and Shear, 2010). The overall approach to practice can be categorized as being primarily aimed at either the loss of object in the self, the loss of self in object, or the loss of both self and object, though if followed far enough, most end up in some type of MPE, irrespective of how it may be interpreted after the fact (Guenther, 1977; Dunne, 2015).

Meditative absorption states have been mapped based on the altered states of consciousness they induce or based on the disappearance or appearance of other phenomenal contents (Lama, 1980; Aranya, 1984). For example, altered states resulting from the traditional mindfulness practice have been categorized into four lower absorption states (Pali: jhana) and described primarily as pervasive sensory-affective states of pleasure, joy, contentment, and peace, while the four higher jhanas are described as more refined mental states of boundlessness of space, consciousness, no-thingness, and neither perception nor non-perception (Shankman, 2008). The end point of this practice is an absorption state described as temporary cessation of all perception, feeling, and consciousness. Whether any phenomenal content remains in such a state has been debated (Sharf, 2013; Srinivasan, 2020). In the Tibetan practices of six yogas, the progressive absorption stages are characterized by the appearance of different inner light visions and the cessation of corresponding conceptual mentation. The end point is conceived of as passing beyond cessation, to the isolated clear light of awareness, free of any other phenomenal content (Tsongkhapa, 1996; Varela, 1997). This then is an episode of isolated NDA, and, similar to types of lucidity during deep sleep, it can be regarded as a MPE with explicitly present NDA.

While these two examples of full absorption come from closely related traditions, differences in reported absorption experiences are due to more than differences in their respective philosophical outlooks. They have also to do with the differences in methods of practice. In mindfulness, the sustained attention applied at the beginning of practice is increasingly replaced with open monitoring without any breath regulation, so that as jhanas unfold one allows them to arise and subside of their own accord and merely follows them. In the six yogas, one uses sustained attention throughout, while focusing precisely on points in the core of one’s body and applying a breath retention technique.

Most absorption states generated through meditation practice can be experienced in a more dualistic, or in a more unitary way. Within the ordinary subject-object dualistic structure of experience, the meditator as a subject observes the state of reduced phenomenal content as an object upon which he or she is meditating. For example, one can do mindfulness practice while perpetuating a dualistic subject-object split through conceptual labeling or noting of jhanas, in which case the self is still there even though it is implicit. Conversely, they can appear as more unitary with a different degree of absence of dualistic structuring. These differences can be summarized as changes in the dualistic sense of self as the various processes that construct and maintain it are allowed to relax and become quiescent. This progressive attenuation or deconstruction is sometimes referred to simply as forgetting the self (Tanahashi, 2013). Starting with relaxation and silencing of the layers of inner narrative related to autobiographical-relational-social self, it progresses to the cessation of processes specifying phenomenal core self, such as agency, body-ownership, and spatio-temporal located-ness, then to the basic interoceptive sense of body presence (Farb et al., 2007; Siderits et al., 2013; Dahl et al., 2015; Dor-Ziderman et al., 2016; Metzinger, 2020). However, such temporary quieting down in and of itself may not yet be cessation of the constructed self as the implicit categorizations that reify the self and objects may be still operating in the unconscious substrate (Germano and Waldron, 2006). Thus, selflessness can be understood in a more refined way as not merely inner silence but as a more fundamental transformation of the nature of cognition in which cognitive processes switch from the exclusive reliance on conceptually constructed dualistic subject-object structures to a more direct unconstructed way of cognizing *via* NDA itself.

## Nondual Awareness

NDA is a type of awareness, a basic non-conceptual, non-propositional awareness, without subject-object dualistic structure, hence “nondual.” Statements pointing to this awareness can be most frequently found in the Indian and Tibetan nondual traditions, where it is described as:

“the pure element of awareness in all knowing. It shines by its own light; it is self-luminous” (Gupta, 1998, p.18).

“…essence of awareness, empty, lucid, and free of elaborations” (Rabjam, 2001, p.141).

“… an infinitely spacious expanse… unchanging, without transition, spontaneously present, and uncompounded” (Rabjam, 2001, p.51).

### Mirror Metaphor

NDA has traditionally been compared to a mirror, while phenomena that appear to it have been compared to reflections in the mirror (Norbu, 1987; Josipovic, 2016). Its manner of knowing phenomena can be compared to mere reflecting, without categorization or further conceptual elaboration, that is, without associating, evaluating, forming decisions, or taking itself as a reified subject that knows phenomena as objects. Thus, the perceptual, affective, or cognitive contents and the states of arousal that co-occur with NDA are, so to speak, parallel to it, the way that images in a mirror are to the mirror itself. Just as a mirror is unaffected by images reflected in it, the basic properties of NDA remain, in principle, invariant regardless of what and how much phenomenal content is present, irrespective of the level of arousal (above a certain minimum threshold). The level of arousal can be compared in this metaphor to the amount of illumination in the room, so that some minimum amount is necessary for the mirror to reflect anything, but aside from that, changing the level of illumination affects only images in the mirror and not the mirror itself. Since phenomena do not affect NDA; NDA can encompass and pervade any type of content, whether perceptual, affective, or cognitive. It then knows phenomena as essentially not different from itself (Rabjam, 2007). That is, in addition to individual characteristics that make them appear the way they do, phenomena at that time also appear as having the same basic properties or dimensions as NDA. This is the key point that we will return to in the “Discussion” section.

### Dimensions of Nondual Awareness

A number of distinct properties or dimensions of NDA can be self-evident from traditional and contemporary reports (Rabjam, 2001; Rao, 2005; Laish, 2015; Josipovic, 2019). Although a great many labels with overlapping meaning can be used to describe them, different dimensions of NDA have been most frequently subsumed into a few cardinal ones: presence or being, emptiness, inherent reflexivity, luminosity, bliss, and singularity. Presence or being is its all-encompassing existence. Emptiness is both an absence of phenomenal contents other than itself and an absence of conceptual processes that reify awareness as either subject or object and create the dualistic structure of experience. Inherent non-representational reflexivity, traditionally termed self-knowing or self-recognition (Ksemaraja and Singh, 1990; Rabjam, 2001; Laish, 2015), refers to NDA knowing itself to be aware without relying on mediation by conceptual and symbolic representations, hence non-representational. Luminosity or radiance refers to the transparent clear light of awareness’ cognizance that illuminates both itself and any phenomena that may be present to it. Bliss is here the silent contentment of being entirely complete in itself, with no sense of any lack or any need to be other than what it is. Singularity refers to NDA being singular or homogenous and to the spontaneous unity or inseparability of all of its dimensions, rather than it being a product of the combination of dimensions, hence also, uncompounded.

NDA can be regarded to be a special kind of a self, in the sense that it is a singular aware presence that remains self-same (Deutsch, 1973; Rabjam, 2001). At the same time, because it is empty of reified subject and object, it can be regarded as not having or being a self at all. To the ordinary self, NDA appears as an object of sorts, something one might want to experience or as a capacity one might want to have. To NDA, however, the ordinary self and its constituting processes, to the extent that they can be phenomenally accessed, appear as contents within its space. The arguments between self and no-self perspectives have spanned centuries within the Asian nondual traditions and are beyond the scope of this paper (Siderits et al., 2013). In practical terms, they can be very briefly summed up as follows: If NDA is experienced as a self, it is a sign that some, however subtle, conceptualization that reifies awareness as the subject is still present, indicating that the emptiness dimension has not yet been fully realized (MacKenzie, 2012). Alternatively, if NDA is not realized as who one is, then it is still being reified as a subtle object upon which one is meditating, indicating that the dualistic split between meditator and awareness has not yet collapsed and the nondual nature of awareness has not been fully realized (Josipovic, 2014).

The main property or dimension of NDA of interest to us here is its inherent reflexive cognizance (Higins, 2011). As previous argued in more detail (Josipovic, 2019), this inherent self-knowing or reflexivity makes NDA a unique kind, in the sense that it differentiates NDA from all other phenomenal contents, as well as from the functions of consciousness like attention, memory, and so on, and from the various states of consciousness caused by the global levels of arousal in the brain. This intrinsic capacity for unmediated self-knowing qualifies this awareness as the consciousness itself or the consciousness-as-such.

Interestingly, since NDA can, in principle, contextualize, or be the space within which any aspect of experience unfolds, including conceptual ones; various self-related contents and self-specifying processes can be present or co-emergent with NDA without necessarily creating the dualistic split in the structure of experience. Which specific self-related contents manifest within NDA at any given time can to a large degree depend on one’s *a priori* commitment to views about self and selflessness. It can also depend on a given situation and the responses it elicits and on the depth and stability of one’s realization of NDA.

Thus, NDA is the space in which experiences unfolds, but there is nothing abstract about this space, as once NDA has been realized, it is the basic space of phenomena (Rabjam, 2001). When NDA is co-occurring with an experience of minimal phenomenal content or full absorption, it can be termed “isolated nondual awareness,” after traditions in which progressive stages of absorption are used to isolate this awareness from other phenomenal contents (Tsongkhapa, 1996). How phenomenal content appears within NDA can be termed nonduality. Two mistaken conclusions occur due to an incomplete understanding of the relationship between NDA and phenomenal content. The first one is that for NDA to be present, phenomenal content has to be minimal, that is, one needs to be in full absorption. This is not so, since NDA is, in a sense, orthogonal to any content. The second one is that, if NDA is present during wakeful experience, there cannot be any conceptual processes occurring at the same time. This is not accurate because NDA can co-occur with any content, whether perceptual, affective, or cognitive, which appears in it, so to speak, like an image in a mirror.

## Discussion

The majority of meditation methods involve cultivation of constructed states that depend on either decreasing or increasing levels of arousal and enhancing tonic or phasic alertness, respectively (Gellhorn and Keily, 1972; Aranya, 1984; Young and Taylor, 1998; Wallace, 2006; Craigmyle, 2013; Amihai and Kozhevnikov, 2014, 2015). In contrast, meditation methods that are concerned with NDA and authentic being, do not involve manipulating the arousal level, since NDA is independent from it, and can be discovered and present whether the arousal level is low or high, whether tonic or phasic alertness is dominant (Rabjam, 2001; Rabjam and Thondup, 2002). Such meditation methods usually carry a warning not to mistake NDA for a constructed or altered state of consciousness (Manjusrimitra and Lipman, 2001). Even though contemplative traditions sometimes refer to NDA as basic wakefulness, this does not mean that NDA is itself a specific state of arousal, like being awake, as it can be present and self-recognized whether one is fully awake or asleep in lucidity during dream or dreamless sleep. Granted, ordinary lucidity in either dreaming or dreamless sleep is not in and of itself the NDA, as a usual lucidity is the functioning of ordinary dualistic conceptual mentation. However, even such lucidity is by no means a hallucination or a mistaken assessment of the state one is in. Once realized, either within the usual waking state or when isolated in full absorption, NDA can be also realized within the dreaming and dreamless sleep (Tsongkhapa, 1996; Wangyal, 1998). NDA is also not a simple or bare wakefulness, because there are instances of bare wakefulness without any awareness (Boly et al., 2012). Likewise, conscious wakefulness (Metzinger, 2020) still has two different elements in it: a level of arousal experienced as wakefulness, and the awareness itself, in addition to whatever phenomenal contents may be present. In sum, since NDA can, in principle, co-occur with any amount of arousal, from a minimal level necessary for it to function to some maximal level of hyper-arousal that can occur in altered states of consciousness, the level of arousal does not specify NDA.

Various absorption states, including MPEs, can be specified, among others, by the amount of remaining phenomenal content, but NDA cannot be specified in this way, because it can be explicitly present or not with any amount of phenomenal content. Thus, the term MPE does not adequately capture what NDA, or consciousness-as-such, is.

Furthermore, it has been suggested that the presence of any phenomenal content however minimal implies a representational state (Metzinger, 2020). The perspective presented here disagrees with this and is closer in spirit to the phenomenological notion of pre-reflective awareness. Because NDA knowing itself is its inherent property, and does not constitute a transitive relation, there is no need for a representation, irrespective of whether NDA is isolated from other phenomenal content or co-occurring with it. Granted, using the term non-representational reflexivity for this knowing can be confusing, as reflexivity usually indicates a second-order representational cognition, but the term reflexivity as applied here indicates a very different kind of knowing, a non-representational, non-transitive reflexivity. Likewise, describing the activation of this inherent reflexivity as self-recognition does not imply recognition *via* category matching (Ksemaraja and Singh, 1990; Josipovic, 2019) The awareness-of-awareness practice, found in some contemplative traditions, has a relational representational structure as long as NDA has not recognized or realized itself. Once activated, this inherent non-representational self-knowing is just an essential property of NDA (Josipovic, 2019). The term awakened mind, as used in nondual contemplative traditions, does not refer to a state of wakefulness but to this activating of the inherent reflexivity by which awareness knows itself innately (Rabjam, 2001).

To the extent they are consciously accessible, various mental representations can appear to NDA as contents within its space, but they are parallel or orthogonal to it, so to speak, and do not define it. Furthermore, similar to the pre-reflexive perception, NDA is aware of any thoughts and other internal experiences as a first-order non-representational knowing, instead of as a second-order representational one, in other words, as just witnessing or mirroring without representation^1^. The significance of this non-representational view for our discussion is that NDA or consciousness-as-such is not a representation or a model of arousal, even though such a model may exist and function to optimize wakefulness.

When the term MPE is expanded to include the properties or dimensions of NDA, there can be considerable overlap in what the two terms denote, and the difference between them may become difficult to appreciate (Metzinger, 2020). However, as properties of NDA are non-representational and inherent to it, and are not states constructed through representations, these only appear to be the same, without actually being so. Likewise, once realized, NDA functions as the all-pervading non-conceptual space (Sansk. Dharmadhatu) that contextualizes both the intrinsic and the extrinsic aspects experience into one unified whole (Rabjam, 2007; Josipovic, 2014). This space is NDA itself, not a representation of arousal or wakefulness, irrespective of how abstract this representation may be.

As discussed in the preceding section, since phenomena, in principle, do not affect NDA, NDA can encompass and pervade any type of content, whether perceptual, affective, or cognitive. Within the space of NDA, phenomena, in addition to the individual characteristics that make them appear the way they do, also appear as having the same basic properties or dimensions as NDA. In other words, as NDA reflects phenomena that appear to it, those phenomena and states reflect the properties of NDA. Properties attributed to MPEs like epistemicity, cognizance, reflexivity, self-luminance, etc. (Metzinger, 2020) can then be seen as reflections of the inherent non-representational properties of NDA, that can appear either as unmediated or as meditated *via* mental representations.

NDA has also been contrasted with the substrate consciousness, a neither fully conscious nor entirely non-conscious matrix that is thought to function as a pervasive potential for memory and structuring of experience (Germano and Waldron, 2006). The substrate is unconscious in the sense that it is constituted by the failure of NDA to recognize itself, an obscuration of awareness’ inherent reflexivity, and by the subsequent conceptual reification of NDA as a subject and of other phenomenal contents as objects, which are ordinarily unconscious processes. The substrate stores patterns of organizing experience along this dualistic subject-object polarity, from basic propositional beliefs and categorizations, to elaborate self-world models (Higins, 2011; Wallace, 2018). The purpose of contrasting NDA with the substrate is to emphasize that a mere reduction in the amount of phenomenal content or in the level of arousal does not qualify a state as being NDA or consciousness-as-such, because, until NDA has self-recognized, all experiences, no matter how minimal, have the substrate as their basis (Germano and Waldron, 2006; Higins, 2011; Wallace, 2018).

By further inflating the meaning of the term MPE, a great number of states with differing amounts phenomenal content, such as, internal silence, engagement in sequential steps of meditation techniques, or states of flow-like absorption in perceptions and actions, can all be brought under the umbrella of MPE (Metzinger, 2020). An ongoing silent alert state of vigilance and monitoring or mindfulness, with properties similar to those of NDA, can be experienced as either the background or the foreground context of such states. But without NDA self-recognizing, neither these states nor their context, can pass beyond the substrate. The question then becomes: Who or what is being mindful? Or, who or what is aware of any specific property? Realizing this requires, at first, to turn the attentional focus all the way around to face the awareness itself, and then, to sustain it there while not accepting any conceptual solutions for an answer, even when they agree with the established teachings of one’s tradition or with one’s personal beliefs. Instead, the holding of the question with intention to realize or directly experience the nature of awareness, as one persists in awareness-of-awareness, will lead to activating of the inherent non-representational self-knowing of NDA and the revealing of NDA as the “innermost essence” of consciousness (Rabjam, 2001).

## Conclusion

Using the famous Zen ox-herding pictures as a metaphor, we can, arguably, compare the current state of consciousness research to being stuck in stage two of the path of discovering “the Ox”: Recent advances in the scientific, philosophical, and psychological research on consciousness have given us a plethora of data that, like seeing traces of the Ox on the ground, stimulate our collective imagination and theorizing. But somehow, despite all those, we are not yet seeing the Ox itself. Current resurgence of interest in MPEs could be an important contribution to this search. However, it is crucial that we do not confuse the phenomenal contents or the levels of arousal, and their representations and models, for consciousness-as-such. Here, we have argued that only NDA, a basic non-conceptual, non-propositional awareness, that is a unique kind, can truly be considered to be consciousness-as-such. Further research is needed to advance our understanding of this foundational aspect of consciousness. Along the way, we need to resist the temptation to reduce it to other, better understood, aspects of consciousness.

## Author Contributions

ZJ wrote introduction, NDA and discussion sections. VM wrote introduction and MPE sections. Both authors revised all sections.

### Conflict of Interest

The authors declare that the research was conducted in the absence of any commercial or financial relationships that could be construed as a potential conflict of interest.

## Footnotes

^1^We thank Aviva Berkovich-Ohana for this comparison.
